# High linear-energy-transfer radiation can overcome radioresistance of glioma stem-like cells to low linear-energy-transfer radiation

**DOI:** 10.1093/jrr/rrt095

**Published:** 2013-08-16

**Authors:** Yuki Hirota, Shin-Ichiro Masunaga, Natsuko Kondo, Shinji Kawabata, Hirokazu Hirakawa, Hirohiko Yajima, Akira Fujimori, Koji Ono, Toshihiko Kuroiwa, Shin-Ichi Miyatake

**Affiliations:** 1 Department of Neurosurgery, Osaka Medical College, 2–7 Daigaku-machi, Takatsuki City, Osaka 569-8686, Japan; 2 Particle Radiation Oncology Research Center, Research Reactor Institute, Kyoto University, Osaka, Japan; 3 Cellular and Molecular Biology Team, National Institute of Radiological Science, Heavy-ion Radiobiology Research Group, Research Center for Charged Particle Therapy, Chiba, Japan

**Keywords:** glioblastoma multiforme, glioma stem cells, linear energy transfer, neutron beams, gamma rays

## Abstract

Ionizing radiation is applied as the standard treatment for glioblastoma multiforme (GBM). However, radiotherapy remains merely palliative, not curative, because of the existence of glioma stem cells (GSCs), which are regarded as highly radioresistant to low linear-energy-transfer (LET) photons. Here we analyzed whether or not high-LET particles can overcome the radioresistance of GSCs. Glioma stem-like cells (GSLCs) were induced from the GBM cell line A172 in stem cell culture medium. The phenotypes of GSLCs and wild-type cells were confirmed using stem cell markers. These cells were irradiated with ^60^Co gamma rays or reactor neutron beams. Under neutron-beam irradiation, high-LET proton particles can be produced through elastic scattering or nitrogen capture reaction. Radiosensitivity was assessed by a colony-forming assay, and the DNA double-strand breaks (DSBs) were assessed by a histone gamma-H2AX focus detection assay. In stem cell culture medium, GSLCs could form neurosphere-like cells and express neural stem cell markers (Sox2 and Musashi) abundantly in comparison with their parental cells. GSLCs were significantly more radioresistant to gamma rays than their parental cells, but neutron beams overcame this resistance. There were significantly fewer gamma-H2AX foci in the A172 GSLCs 24 h after irradiation with gamma rays than in their parental cultured cells, while there was no apparent difference following neutron-beam irradiation. High-LET radiation can overcome the radioresistance of GSLCs by producing unrepairable DNA DSBs. High-LET radiation therapy might have the potential to overcome GBM's resistance to X-rays in a clinical setting.

## INTRODUCTION

Radiation therapy with surgery and chemotherapy is the standard treatment for glioblastoma multiforme (GBM) [1]. However, the prognosis of patients with GBM has not improved in recent decades, and almost half of GBM patients do not survive the first year after diagnosis. Thus, another, more promising therapy for GBM is needed. Recently, some reports have shown the presence of glioma stem cells (GSCs) in malignant gliomas [2–4]. These cells are highly resistant to radiotherapy because of their enhanced checkpoint response to radiation [5]. Other studies have shown that GSCs express high levels of sirtuin family genes (especially the SirT1 gene) and that these upregulations are relevant to radiosensitivity because they modulate apoptotic activity in response to irradiation to GSCs [6]. As a result, GSCs are now known to play important roles in tumor progression and relapse after radiotherapy and chemotherapy, and new therapeutic strategies targeting GSCs should be developed to treat patients with GBM. In the previous reports, radioresistance of GSCs was studied in a subpopulation with a specific phenotype. In these studies, it was difficult to use appropriate control cells for the GSCs. Therefore, we induced glioma stem-like cells (GSLCs) in which the phenotypes of GSCs were enriched, and used the wild-type GBM cells as controls in this study.

On the other hand, we have applied boron neutron capture therapy (BNCT) for malignant brain tumors, including GBM [7–9]. This is a unique tumor-selective particle radiotherapy using neutron irradiation, especially thermal neutron irradiation. Boron-10 (^10^B) releases alpha (^4^He) and ^7^Li particles through ^10^B (n,α)^7^Li reaction. The key players in the anti-tumor effects of BNCT are these high linear-energy-transfer (LET) particles. With BNCT, good results have already been achieved for patients with newly diagnosed GBM and recurrent malignant glioma [9, 10], although the numbers of such cases in clinical trials have been limited.

So far, the radioresistance of GSCs has been examined mainly in terms of low-LET radiation such as X-rays or gamma rays. Therefore, we hypothesized that high-LET radiation could overcome the radioresistance of GSCs. In fact, a previous study showed that high-LET radiation was more effective than low-LET radiation for promoting DNA damage [11]. Here, we employed a reactor neutron-beam irradiation system that produces high-LET proton particles through elastic scattering and nitrogen capture reaction. We analyzed the usefulness of high-LET radiation for overcoming the radioresistance to low-LET radiation in GSCs using GSLCs, as well as the ability of these cells to recover from radiation-induced DNA damage by a gamma-H2AX assay.

## MATERIALS AND METHODS

### Cell culture

The human GBM cell line A172 was purchased from American Type Culture Collection (Manassas, VA) and cultured in Dulbecco's modified Eagle's medium (DMEM; Invitrogen, Carlsbad, CA) with 10% fetal bovine serum (FBS) with penicillin and streptomycin at 37°C in an atmosphere of 5% CO_2_. GSLCs were induced from A172 cells in serum-free medium (SFM) as described previously [12]. The SFM was composed of DMEM/F12 (Sigma-Aldrich, St Louis, MO), 20 ng/ml basic fibroblast growth factor (Peprotech, Rocky Hill, NJ), 20 ng/ml epidermal growth factor (Peprotech), 2 µg/ml heparin (Sigma-Aldrich), and B27 supplement (50×; Life Technology/Invitrogen).

### Western blot analysis

Cells were cultured for 7 d in each culture medium. Protein samples were prepared with 10% sodium dodecyl sulfate-polyacrylamide gel electrophoresis and transferred onto nitrocellulose membranes. Immune complexes were formed by incubation with the stem cell markers CD133 (Cell Signal Technology, Danvers, MA), Sox2 (Cell Signal Technology), and Musashi (Cell Signal Technology) overnight at 4°C. As a control for the housekeeping gene products, Ku70 (Thermo Scientific, Waltham, MA) was employed. Blots were washed and incubated for 1 h with horseradish peroxidase-conjugated anti-mouse and anti-rabbit secondary antibodies (Santa Cruz Biotechnology, Santa Cruz, CA). Immunoreactive protein bands were detected by using an enhanced chemoluminescence Advance Western Blotting Detection Kit (GE Health Care, Buckinghamshire, UK), and Image Reader LAS-1000 Pro ver. 2.5 (Fuji Photo Film, Tokyo, Japan).

### Fluorescence-activated cell sorting analysis

Cells were cultured for 7–14 d in each culture medium. Cells were collected and incubated with anti-CD133 antibody (Bioss, Woburn, MA) for 1 h at 37°C. After washing, the cells were incubated with Alexa Fluor 647-labeled anti-rabbit secondary antibody for 30 min at 37°C, then analyzed by fluorescence-activated cell sorting (FACS) using a BD FACS Aria Cell Sorter (BD Bioscience, San Jose, CA).

### Gamma-ray and neutron-beam irradiation

Two sets of A172 cells, one cultured with serum-containing medium (DMEM + 10% FBS) and the other cultured with SFM, were trypsinized, and single-cell suspensions were placed into a Teflon tube and irradiated at room temperature by neutron beams or gamma rays.

At the Heavy Water Column of the Kyoto University Research Reactor (KUR), neutron-beam irradiation was performed at a power of 1 MW. The neutron fluence was measured from the radioactivation of gold foil. Contaminating gamma rays, including secondary gamma rays, were measured with thermoluminescence dosimeter (TLD) powder. The TLD used was beryllium oxide (BeO) enclosed in a quartz glass capsule. BeO itself is sensitive to thermal neutrons [13]. The average neutron fluxes were 1.0 × 10^9^ n/cm^2^/s for the thermal neutron range (less than 0.6 keV), 1.6 × 10^8^ n/cm^2^/s for the epithermal neutron range (0.6–10 keV), and 9.4 × 10^6^ n/cm^2^/s for the fast neutron range (more than 10 keV). The total absorbed doses resulting from fast, epithermal, and thermal neutron-beam irradiation were calculated as the sum of the absorbed doses attributed primarily to ^1^H(n,n)^1^H, ^14^N(n,p)^14^C, and contaminating gamma rays. The dose-converting coefficients and details of the calculation method have been described previously [14, 15].

Gamma-ray irradiation was applied using a ^60^Co gamma-ray irradiator at a dose rate of 1.3 Gy/min.

### Colony-forming assay

Cell survival was defined using a colony-forming assay. The irradiated cells were seeded into 100 mm dishes at various densities depending on the physical dose that cells received, and cultured in a serum-containing medium. After 13–15 d, the colonies were stained with methylene blue. A cell cluster containing at least 50 cells was considered a single colony. The surviving fraction was calculated as the number of colonies of treated cells divided by that for the control cells. The D_10_ values were derived by linear quadratic model analysis for cell survival curves. The relative biological effectiveness (RBE) for neutron beams was obtained as the ratio of the mean value of D_10_ to that of gamma rays.

### Gamma-H2AX focus assay

A Gamma-H2AX focus assay was performed to detect DNA double-strand breaks (DSBs) [16]. Cells were poured onto 22 × 22 mm coverslips in 35 mm dishes filled with medium and placed in an incubator for the stated repair time after irradiation. Briefly, cells were fixed with 4% paraformaldehyde in phosphate-buffered saline (PBS), permeabilized for 10 min on ice in 0.5% Triton X-100 in PBS, washed thoroughly with PBS, and then blocked for 1 h with 3% skim milk in PBS. The coverslips were then incubated with an antibody against histone H2AX phosphorylated on serine 139 (Upstate Biotechnology, Lake Placid, NY) for 2 h at 37°C. After incubation with primary antibody, the cells were washed with PBS, and Alexa Fluor 488-labeled anti-mouse IgG secondary antibodies (Invitrogen) were added. The coverslips were incubated for 1 h at 37°C, washed with PBS, and sealed onto glass slides with 0.05 ml PBS containing 10% glycerol (Wako, Osaka, Japan) and 20 µg/ml DAPI (4'6-diamidino-2-phenylindole; Invitrogen). The cells were examined using a Keyence fluorescence microscope (Keyence, Osaka, Japan), and the green intensity of the phosphor-H2AX signal on digitized images was automatically analyzed using the software package Dynamic Cell Count (Keyence). Using this software package, the numbers and sizes of foci exhibiting high-intensity staining with gamma-H2AX (green) in each type of A172 cell population were determined in more than 100 areas per condition.

### Statistical analysis

Values are presented as means ± standard errors. Statistical analyses were performed using the unpaired, two-tailed Student's *t*-test. A significance level of *P* < 0.05 was used for all analyses. The data on cell survival were fitted to the linear-quadratic dose relationship.

## RESULTS

### Detection of stemness in GSLCs

**Fig. 1.**
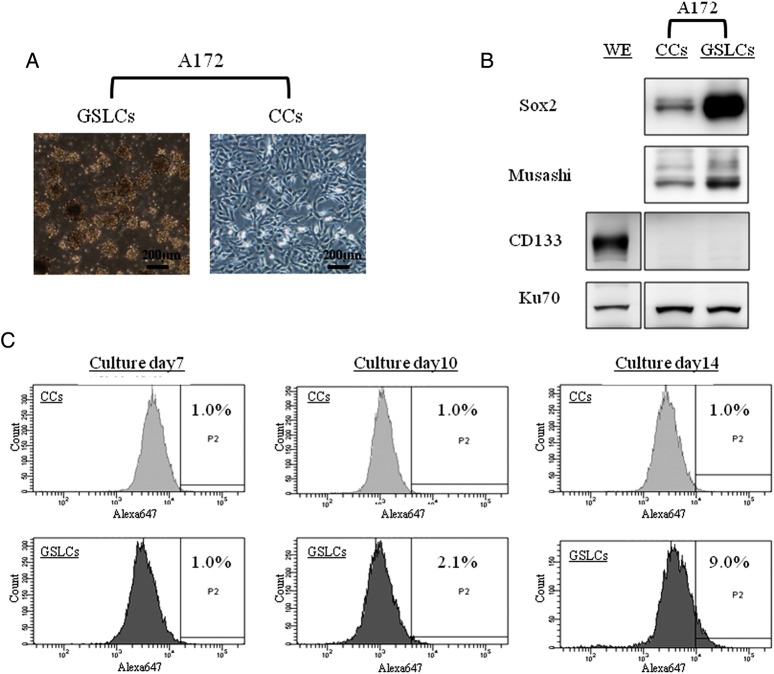
Characteristics of the glioma stem-like cells. (**A**) The morphology of human glioma cell line A172 cultured for 7 d in serum-containing medium or serum-free medium. (**B**) The expression of typical stem cell marker proteins as examined by Western blot assays on Day 7 after culture. (**C**) The ratio of CD133-positive cells in FACS analysis; the number of days of culture is shown in each column, and the rate of CD-133-positive GSLCs was measured with a cutoff value obtained from the fluorescence intensity that occupied 1% by putative CD133-positive CCs in the total population. GSLCs = glioma stem-like cells; CCs: control cells; WE = WERI-Rb-1 (the retinoblastoma cell line used as a positive control for anti-CD133 Ab).

Figure 1 shows the characteristics of the GSLCs. To induce GSLCs, we cultured the A172 cells in SFM, as described above. Seven days after culturing in SFM, these cells were form-floating, neurosphere-like spheroid cells (Fig. 1A). In the Western blotting analysis, we found that two neural stem cell markers, Sox2 and Musashi, were more highly expressed in the GSLCs than in the A172 cells cultured in serum-containing medium as control cells (CCs) (Fig. 1B). However, no apparent CD133 expression was detected in either GSLCs or CCs that were cultured for 7 d. Therefore, we changed the CD133-detection assay for FACS analysis by using several time-points. In the FACS analysis, the ratio of CD133-positive GSLCs increased by 9% after 14 d, whereas the ratio of CD133-positive CCs was unchanged (Fig. 1C). The FACS analysis confirmed marked positivity in the WERI-Rb-1 (WE) cells, a retinoblastoma cell line used as a control (data not shown).

### Radiosensitivity of GSLCs and CCs

**Fig. 2.**
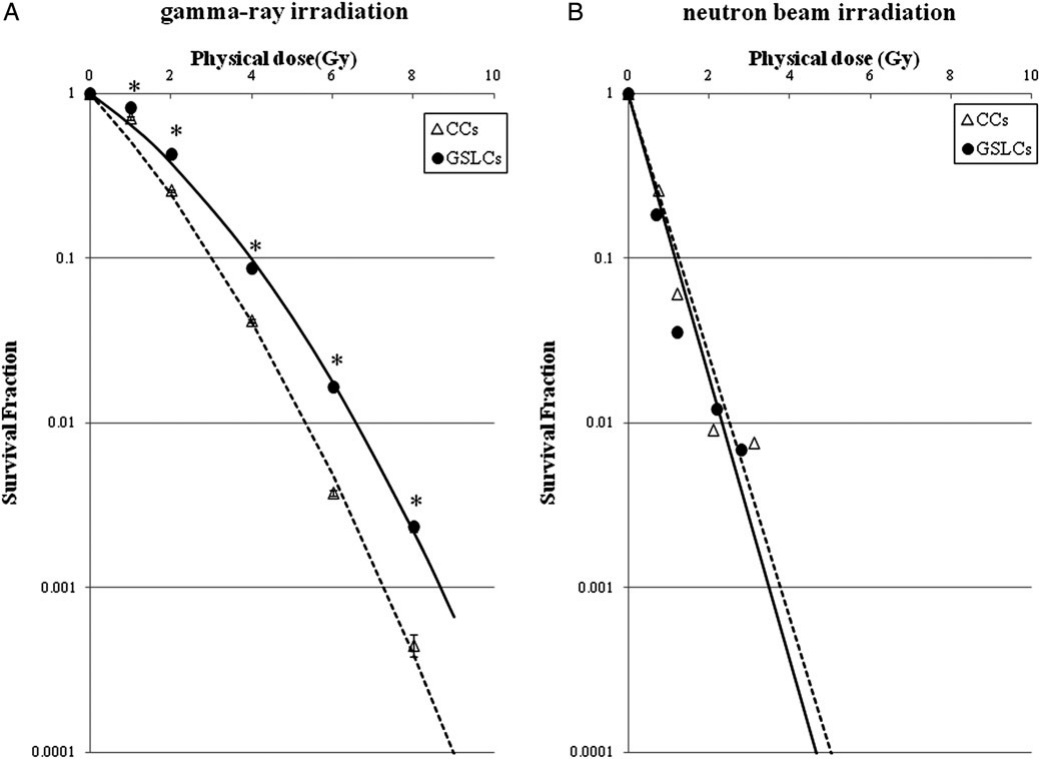
Cell survival curves of GSLCs induced from A172 cells cultured with serum-free medium and CCs cultured with normal medium after gamma-ray (**A**), or neutron-beam irradiation (**B**). The data are fitted with a linear quadratic model. Bars represent the standard errors based on three independent experiments. *P < 0.05 compared with the survival fraction of GSLCs and CCs. GSLCs = glioma stem-like cells; CCs = control cells.

The radiosensitivity of GSLCs was compared with that of CCs under gamma-ray or neutron-beam irradiation. Figure 2 shows the surviving fractions of A172 under the two culture conditions after gamma-ray or neutron-beam irradiation. After gamma-ray irradiation, GSLCs showed significantly greater radioresistance than CCs. On the other hand, after neutron-beam irradiation, there was no significant difference in the sensitivity between GSLCs and CCs. The D_10_ values were calculated by linear regression analysis from the survival curves shown in Fig. 2, and the D_10_ dose parameters for survival following irradiation and their RBEs are listed in Table 1. The D_10_ value represents the radiation dose that produces a survival fraction of 10%. To examine the difference in radiosensitivity between GSLCs and CCs, we referred to the resistance ratio. This ratio was calculated from the D_10_ dose of GSLCs per that of each respective CC by these two forms of irradiation. For example, under gamma-ray irradiation, the ratio of the D_10_ dose of GSLCs to that of CCs was 3.98/3.02 = 1.318. On the other hand, under neutron-beam irradiation, the D_10_ dose of GSLCs per that of CCs was 1.17/1.25 = 0.936. The resistance ratio of neutron beams was smaller than that of gamma rays. Consequently, neutron-beam irradiation overcame the resistance to gamma-ray irradiation in A172 GSLCs. In other words, these results suggested that A172 GSLCs, which were radioresistant to gamma rays, became sensitive to neutron beams.

**Table 1.** D_10_ physical dose and RBE (relative biological effectiveness)

	Irradiation	
(CCs)	gamma rays	neutron beams
D_10_ physical dose	3.02	1.25
RBE^*a*^		2.42
**(GSLCs)**		
D_10_ physical dose	3.98	1.17
RBE		3.40
**Resistance ratio**^***b***^**(GSLCs/CCs)**	1.318	0.936

^*a*^The ratio of the D_10_ physical dose compared to that of gamma rays. ^*b*^The ratio of the dose of radiation necessary to obtain the D_10_ endpoint from GSLCs to that necessary in CCs. GSLCs = glioma stem-like cells, CCs = control cells, D_10_ = the radiation dose that produces a surviving fraction of 10%.

### Persistence of gamma-H2AX foci following irradiation

**Fig. 3.**
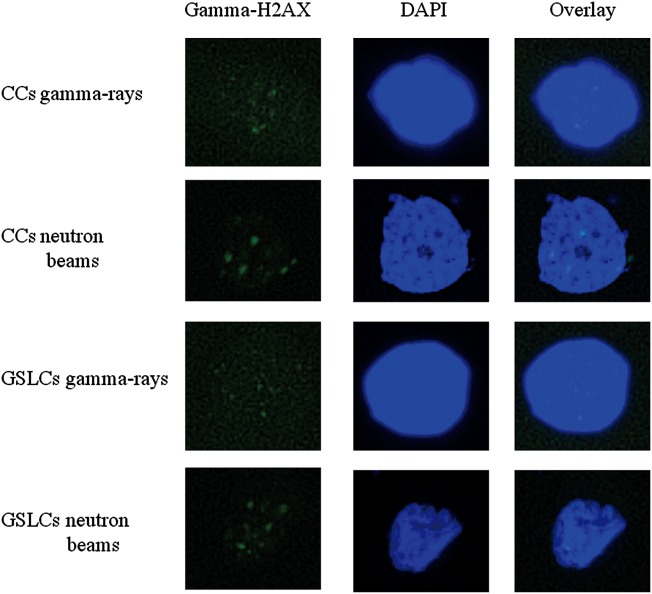
Representative images of nuclear gamma-H2AX foci of CCs and GSLCs in A172. These cells were irradiated with different types of beams (total physical dose = 4 Gy) and fixed at 24 h post-irradiation for gamma-H2AX detection. DAPI = staining of nuclear DNA; Gamma-H2AX = staining of gamma-H2AX foci; GSLCs = glioma stem-like cells; CCs = control cells.

**Fig. 4.**
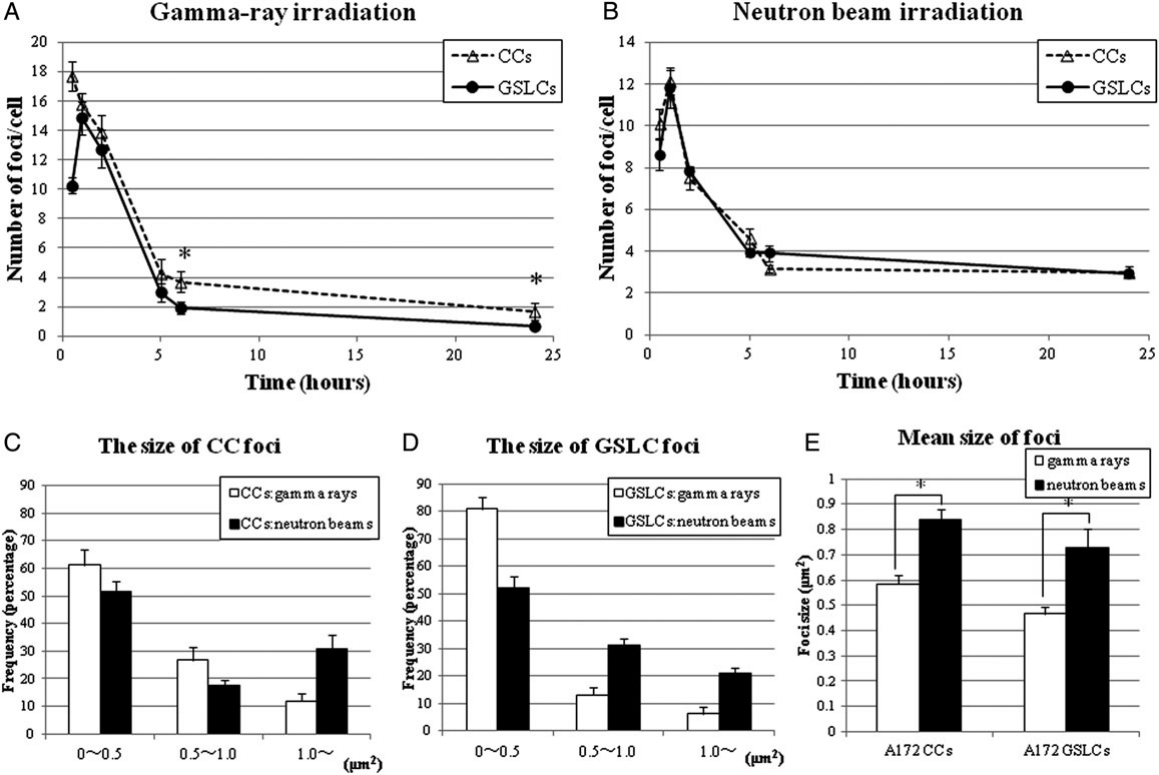
Change in the number of induced nuclear gamma-H2AX foci and the histograms of gamma-H2AX foci size, at the times indicated post-irradiation in A172. These cells were irradiated with different types of beams (total physical dose = 4 Gy). (**A**) and (**B**) The numbers of gamma-H2AX foci per cell of GSLCs and CCs in A172 after the different types of radiation. (**C**) and (**D**) Distribution of gamma-H2AX foci sizes for A172 at 24 h post-irradiation. (**E**) Mean gamma-H2AX foci size for each type of A172 cells at 24 h post-irradiation. Bars represent the standard errors. *P < 0.05 compared with gamma-H2AX foci per cell in GSLCs and CCs. GSLCs = glioma stem-like cells; CCs = control cells.

Figure 3 shows representative images of each type of A172 cells at 24 h after each type of irradiation. The fluorescence intensity of gamma-H2AX foci produced by neutron beams was stronger than that produced by gamma rays in both GSLCs and CCs, under the same staining conditions and the same photographic exposure time (Fig. 3). At a glance, the foci in both CCs and GSLCs produced by neutrons seemed larger than those produced by gamma rays. Figure 4A and B show the change in the numbers of gamma-H2AX foci following 4 Gy of gamma-ray or neutron irradiation in GSLCs and CCs induced from A172 cells. There were significantly more gamma-H2AX foci per cell in CCs than in GSLCs 24 h after gamma-ray irradiation. However, after neutron-beam irradiation, there was no apparent difference between GSLCs and CCs in the number of gamma-H2AX foci. Figure 4C and D show the distribution histograms of the size of foci induced in GSLCs and CCs, respectively, and Fig. 4E shows the mean size of gamma-H2AX foci at 24 h post-irradiation, measured using the BZII image analysis system (Keyence). Figure 4C, D and E reveal definitively that neutron-beam irradiation induced larger gamma-H2AX foci than those observed after gamma-ray irradiation, not only in CCs but also in GSLCs of A172 cells. These results might suggest that DSBs were repaired more efficiently in GSLCs than in CCs following gamma-ray irradiation. In contrast, under neutron irradiation, the DNA DSBs were not repaired efficiently in either GSLCs or CCs.

## DISCUSSION

Research on GSCs has been conducted for many years, and GSCs have been found to contribute to the recurrence and resistance to therapy of malignant gliomas [2–6]. The difficulty of treating GBM may be attributed to the existence of GSCs in GBM, judging from the numerous published findings about GSCs.

In previous reports, GSCs were isolated from glioma tissues as spheres cultured in SFM containing stem-cell mitogens, epidermal growth factor and fibroblast growth factor, which is the same method used to isolate neural stem cells from brain tissue [2–4, 17]. Because of the lack of serum and the low plating density, most of the cells die, except those that divide in response to the stem-cell mitogens. The growth-factor-responsive cells proliferate to form floating clusters called neurospheres [18]. In this study, we induced GSLCs from cells of the human GBM line A172 using the same isolation-GSCs method as described previously [12]. In SFM containing the stem-cell mitogens, GSLCs were produced as neurosphere-like spheroid cells, and expressed neural stem cell markers such as Sox2 and Musashi (Fig. 1A and B) on Day 7 after induction. Actually, CD133 was hardly detected in Western blot analysis after 7 d of culture. Therefore, we performed FACS analyses and determined the ratio of CD133 positivity between GSLCs and CCs by kinetics study. The CD133-positive fraction in GSLCs increased gradually in comparison with that in CCs day by day, and on Day 14, 9% of GSLCs were CD133-positive, although many GSLCs were still negative for CD133. In addition, 30 d of induction culture resulted in a higher percentage of CD133-positive GSLCs—up to 21% (data not shown). We speculate that it took a long time for CD133-positive cells to be refined in the SFM, and thus there was an insufficient number of CD133-positive cells for detection by Western blot analysis on Day 7. Indeed, CD133 positivity in our GSLCs from A172 on Day 7 was still small in number, but other stemness markers increased compared with CCs, and CD133 is not always a good GSC marker [19–21]. In addition, GSLCs induced by this method showed the upregulation of ATP-binding cassette transporter G2 and increased chemo-resistance in comparison with CCs (data not shown and manuscript in preparation). Above all, these GSLCs from A172 were somewhat radioresistant for low-LET gamma rays. Thus, we judged that these GSLCs were adequate for our further experiments. In any event, GSLCs had some degree of stemness. Actually, we tried to induce GSLCs from three GBM lines. Among them, GSLC from A172 was most prominent with GSC phenotype and apparent radioresistance to low-LET γ-rays. Thereafter, we used GSLCs from A172 in the current studies. We assessed the radiosensitivity of GSLCs using colony-forming assay on Day 7. Although, there might be a possibility of change in radiosensitivity associated with change of expression of CD133, especially in the later period of the induction of GSLCs, such as on Day 28, in the previous report [22], A172 CCs did not express CD 133, while radiation-induced GSLCs of A172 cells did express CD133. This is in accord with our experiment.

To evaluate the difference in radiosensitivity between GSLCs and CCs, we irradiated these cells with gamma rays or neutron beams, and found that the latter could overcome the radioresistance of GSLCs to gamma rays (Fig. 2 and Table 1). To obtain neutron beams, we used the Heavy Water Column of the KUR. These neutron beams consisted of fast, epithermal and thermal neutrons. Each neutron beam produced proton particles by elastic scattering (^1^H(n,n)^1^H) or nitrogen capture reaction (^14^N(n,p)^14^C) at irradiation, and these particles exhibited high-LET radiation. The LET of proton particles produced by the former reaction was about 50 keV/μm, and that produced by the latter reaction was about 35 keV/μm, whereas the gamma rays exhibited low-LET radiation. Therefore, it can be concluded that high-LET radiation can better overcome the radioresistance of GSLCs in comparison with low-LET irradiation. Ionizing radiation produces a broad spectrum of molecular lesions in DNA, including single-strand breaks, DSBs, and a great variety of base damages. DSBs are the most toxic form of DNA damage, because a single unrepaired DSB can lead to abnormal mitosis with losses of large fragments of DNA [23]. Further, it is generally accepted that high-LET radiation induces more serious DNA DSBs than low-LET radiation [11, 24]. In the current study, we demonstrated that high-LET radiation could damage GSLCs that were resistant to low-LET gamma rays. As previously described, GSCs have a large capacity to repair DSBs induced by low-LET radiation [5]. However, it was uncertain whether or not high-LET radiation could cause serious DSBs that were unrepairable, even in GSCs.

To clarify the response to DNA DSBs induced by gamma rays or neutron beams, we employed a gamma-H2AX assay. From a previous report, we judged the persistence of gamma-H2AX foci 24 h after treatment as unrepairable DSB [25]. GSLCs had a larger restoration capacity for DSBs than CCs after low-LET radiation, but could not repair DSBs sufficiently after high-LET radiation (Fig. 4A and B). Because reduced survival was accompanied by the persistence of DNA damage, as evidenced by the persistence of gamma-H2AX foci after irradiation [26], high-LET radiation could produce persistence of DSBs and induce fatal damage even in GSLCs. In fact, it has been reported that to evaluate gamma-H2AX foci in cells exposed to high-LET radiation, the size of the foci should be considered, since high-LET radiation can cause larger gamma-H2AX foci than low-LET radiation [11, 27]. We therefore investigated not only the numbers of foci but also their size after 24 h irradiation by both types of radiation, and found that high-LET radiation could cause larger gamma-H2AX foci than low-LET radiation in both GSLCs and CCs (Figs. 3, 4C, 4D and 4E). As Fig. 4E shows, high-LET radiation led to significantly larger gamma-H2AX foci than low-LET radiation did, in both GSLCs and CCs. Therefore, it is thought that high-LET radiation could cause more serious DNA DSBs than low-LET radiation, even in GSLCs. In the previous report [28], low-LET irradiation might produce relatively large foci with time. In our experiment we demonstrated that high-LET particles produce larger foci in GSLC than low-LET gamma rays.

Indeed, under both gamma-ray and neutron-beam irradiation, more than half of all gamma-H2AX foci were small (0–0.5 µm^2^). It is speculated that the neutron beams from KUR formed a wide-range beam that included gamma rays and secondary gamma rays. At the absorbed dose of 4 Gy, the compositions of fast, epithermal and thermal neutrons as well as of gamma rays were 25.5%, 2.5%, 22% and 50%, respectively. Almost half of the neutron beam components of the absorbed dose were induced by gamma rays, which could explain why small foci were induced mainly by gamma rays, even under neutron-beam irradiation. As described above, Fig. 3 also shows that the fluorescence intensity of gamma-H2AX foci after neutron irradiation was higher than that after gamma-ray irradiation. This may explain why high-LET radiation causes more intense DNA damage than low-LET radiation. A previous study showed that high-LET radiation, such as that from heavy ion therapy, had several potential advantages over low-LET radiation due to its induction of complex DNA damage that was not easily repaired [29], and may have an advantage over low-LET radiation for cancer stem-like cells [30]. Thus, our data also support the potential for use of high-LET radiation for GSCs.

Heavy ion treatment and BNCT are recognized as forms of high-LET radiation. In a previous report, when chemo-irradiation was combined with carbon ion therapy, the median survival time of GBM patients was 17 months [31]. In another report, BNCT followed by X-ray radiation therapy led to a median survival time of GBM patients of 21.3 months, even without chemotherapy [9]. Although both of these reports involved small numbers of patients, the results suggested that, since these high-LET radiations were effective even for GSCs in a clinical setting, patients could show prolonged survival. At the moment these treatment modalities are still at the clinical trial stage, but they may improve the standard treatment for GBM.

Although various treatments for GBM have been tried, an unfavorable prognosis can be expected with the current standard treatment. In the present study, we demonstrated that high-LET radiation may be able to overcome GSC resistance to low-LET radiation. It is necessary to further investigate the usefulness of high-LET radiation for the control of GSCs. High-LET radiation therapies such as BNCT or heavy ion therapy have very important roles in further treatment for therapy-resistant GBM.

## CONFLICT OF INTEREST

The authors have no conflicts of interest to disclose.

## FUNDING

This work was supported by a Grant-in-Aid for Scientific Research (B) (23390355) from the Japanese Ministry of Education, Culture, Sports, Science and Technology to S-IM.
